# Developing Molecular Signatures for Chronic Lymphocytic Leukemia

**DOI:** 10.1371/journal.pone.0128990

**Published:** 2015-06-05

**Authors:** Edouard Cornet, Agathe Debliquis, Valérie Rimelen, Natacha Civic, Mylène Docquier, Xavier Troussard, Bernard Drénou, Thomas Matthes

**Affiliations:** 1 CHU Caen, Laboratory of Hematology, 14000, Caen, France; 2 University of Caen, Medical School, 14000, Caen, France; 3 Département d’Hématologie, Hôpital de Mulhouse, 68051, Mulhouse, France; 4 Genomics Platform iGE3, University Medical Center, 1211, Geneva, Switzerland; 5 Hematology Service, University Hospital Geneva, 1211, Geneva, Switzerland; 6 Clinical Pathology Service, University Hospital Geneva, 1211, Geneva, Switzerland; University of Zurich, Swiss Institute of Bioinformatics, SWITZERLAND

## Abstract

Chronic lymphocytic leukemia (CLL) is a clonal malignancy of mature B cells that displays a great clinical heterogeneity, with many patients having an indolent disease that will not require intervention for many years, while others present an aggressive and symptomatic leukemia requiring immediate treatment. Although there is no cure for CLL, the disease is treatable and current standard chemotherapy regimens have been shown to prolong survival. Recent advances in our understanding of the biology of CLL have led to the identification of numerous cellular and molecular markers with potential diagnostic, prognostic and therapeutic significance. We have used the recently developed digital multiplexed gene-expression technique (DMGE) to analyze a cohort of 30 CLL patients for the presence of specific genes with known diagnostic and prognostic potential. Starting from a set of 290 genes we were able to develop a molecular signature, based on the analysis of 13 genes, which allows distinguishing CLL from normal peripheral blood and from normal B cells, and a second signature based on 24 genes, which distinguishes mutated from unmutated cases (LymphCLL Mut). A third classifier (LymphCLL Diag), based on a 44-gene signature, distinguished CLL cases from a series of other B-cell chronic lymphoproliferative disorders (n = 51). While the methodology presented here has the potential to provide a "ready to use" classification tool in routine diagnostics and clinical trials, application to larger sample numbers are still needed and should provide further insights about its robustness and utility in clinical practice.

## Introduction

Chronic lymphocytic leukemia (CLL) is the most common leukemia in the Western world. Diagnosis is based on the results of flow cytometric analysis of malignant B cells obtained from peripheral blood, bone marrow, lymph nodes, and other organs. The characteristic phenotype is defined by the combination of several surface markers (CD5, CD19, CD20, and CD23), and the Royal Marsden Hospital (RMH) score is widely used to distinguish CLL from other B-cell chronic lymphoproliferative disorders (B-CLPD) [1]. The clinical course is highly heterogeneous, with some patients dying from their disease within months, while others have a normal life expectancy. Predicting the disease outcome is therefore very helpful in patient management and therapeutic decision-making. Over the past decade, several prognostic markers based on genetic, phenotypic, or molecular characteristics of CLL B cells have thus been added to the original staging systems of Rai and Binet (reviewed by Chiorazzi; 2012) [2]. One of the best-studied markers is the immunoglobulin variable region heavy chain (IgVH) mutation status. In fact, roughly one half of CLL cases exhibit somatically mutated variable heavy chain genes and their presence correlates with a less aggressive clinical course [3],[4]. Specific cytogenetic changes have also been associated with unfavorable outcome (e.g.: deletion 17p) or, alternatively, with improved survival (e.g.: isolated deletion 13q) [5].

At diagnosis, the determination of the correct type of B cell disease associated with a precise outcome prediction currently depends on the interpretation of flow cytometry, cytogenetic and molecular analyses by the corresponding experts, i.e. hematologists, cytogeneticists and pathologists. These methods are costly, and labor- and time-intensive. Cheaper, objective and rapid techniques are therefore warranted. Recently, the company NanoString has developed a new high-throughput RNA expression profiling system (nCounter), which allows the direct digital readout of hundreds of mRNA molecules and their relative abundance using small amounts of total RNA (100 ng), without requiring cDNA synthesis or enzymatic reactions (DMGE; digital multiplexed gene expression) [6]. Several groups have shown high correlation with standard Affymetrix-type profiling and with quantitative RT-PCR, and have also applied this technology to mRNA extracted from Formalin-Fixed Paraffin-Embedded (FFPE) material [7],[8]. DMGE was also applied successfully to the classification of GC and ABC subtypes in diffuse large B cell lymphoma [8]. Our group has observed a high correlation between mRNA measured by DMGE and protein levels in a cohort of acute leukemia patients [9].

Using this technique for the study of a cohort of CLL samples we were able to develop a molecular signature for the diagnosis of CLL (LymphCLL Diag) as well as for the distinction between unmutated and mutated cases (LymphCLL Mut). This analysis is technically simple, can be run in every small hospital, and should be ideal for the use in clinical trials as well as for normal routine diagnosis.

## Material and Methods

### Patients and patient characteristics

Fresh peripheral blood (PB) samples were obtained from 30 patients of the Hematology Services of the Geneva, Mulhouse and Caen hospitals, The Ethics Committee of the Hospital of Geneva as well as of the Hospitals of Caen and Mulhouse have approved this research. Written informed consent was obtained from all patients. All patient data was analyzed anonymously. From each sample white blood cells were either (a) lysed directly in RNA lysis buffer (Qiagen, Venlo, Netherlands) and stored at -80°C, or (b) resuspended in DMSO, stored in liquid nitrogen, thawed for the present study, and then put into RNA lysis buffer (Mulhouse), or (c) lysed in RNA lysis buffer, followed by RNA extraction and storage at -80°C (Caen). For each patient a hematologic work-up was performed and the diagnosis of CLL established according to standard diagnostic guidelines ([10]). Flow cytometric analysis on CLL B cells was done for CD5, CD19, CD20, CD23, CD43, CD200, as well as for CD38 and ZAP70 expression, karyotype analysis for the detection of del17p, del13q, trisomy 12.

The analysis of the IGHV-D-J mutation status was performed on genomic DNA after isolation of leukemic cells on a Ficoll gradient. PCR amplification of *IgH* rearrangements was performed with either family-specific VH leader primers [11] or FR1 primers, using the BIOMED-2 protocol [12]. PCR amplicons were subjected to direct sequencing on both strands. Sequence data were analyzed using the IMGT database and the IMGT/V-QUEST tool (http://www.imgt.org). Only productive rearrangements were evaluated. VH sequences with a germline homology of 98% or higher were considered as unmutated, and those with a homology less than 98% were considered as mutated [13].

All the relevant patient information is presented in S1 Table.

Normal blood samples were obtained from blood donors of the Geneva blood transfusion center. Pure CD19+ B cells were prepared from four Ficoll-enriched normal blood samples using a Selection Kit from Stemcell Technologies, according to manufacturer’s instructions. Purity of the isolated cell populations was verified by flow cytometry with specific anti-CD19 and anti-CD20 antibodies and was >95% in all cases (data not shown).

Additional samples for this study were obtained from the three centers from a series of 51 patients with the following diagnoses: 20 mantle cell lymphoma (MCL), 22 marginal zone lymphoma (MZL) including 8 splenic marginal zone lymphoma (SMZL) with villous lymphocytes, 4 follicular lymphoma (FL), 5 hairy cell leukemia (HCL). RNA from these samples was processed in the same way as from the CLL samples described above.

### mRNA Analysis

For the analysis with the nCounter system either 250 ng of extracted mRNA or mRNA in lysis buffer, corresponding to the equivalent of 10^5^ cells, was used, according to the manufacturer’s protocol (Nanostring H Technologies, Seattle, WA, USA). In brief, 4 μl of cell lysate or extracted mRNA was hybridized with the Nanostring CodeSet overnight at 65°C. Probes for the analysis of 290 different antigens were synthesized by NanoString technologies, including probes for nine normalization genes (S2 Table). After probe hybridizations and NanoString nCounter digital reading, counts for each mRNA species were extracted, analyzed using a homemade Excel macro, and then expressed as counts (molecules of mRNA/ sample), as described previously [14]. The nCounter CodeSet contained two types of built-in controls: positive controls (spiked mRNA at various concentrations to assess the overall assay performance), and negative controls (alien probes for background calculation). Data handling and analysis was performed as described: background correction consisted of the subtraction of the negative control average plus two SD from the original counts. To select adequate normalization genes from the series of nine candidates included in the CodeSet (ACTB, TBP, RPL19, RPLP0, G6PD, ABCF1, B2M, TPT1, RPS23), their relative stability was evaluated using geNorm-method [15]. For the final normalization of the sample values the geometric mean of the counts obtained for the three selected normalization genes (RPL19, RPLP0 and TPT1) was calculated and used as normalization factor.

The technical specificities of the NanoString technology (linearity, reproducibility, sensitivity, etc.) have all been previously described [6],[14],[9].

The data have been submitted to GEO and can be accessed via Access Number GSE66425).

### Establishing a gene list for mRNA analysis

We performed an extensive literature search and extracted a set of 290 genes from published articles and public databases, satisfying one of the following criteria: reported to be overexpressed in normal B cells compared to other blood cells; to be over- or under-expressed in CLL samples compared to normal blood samples; to be over- or under-expressed in CLL samples compared to other B-CLPD (S2 Table). In total, a set of 299 genes (290 genes + 9 normalization genes) was used to study the mRNA profile in 5 normal peripheral blood (PB) samples, 4 purified B cell samples, and 30 samples from patients with CLL.

### Statistical analysis

Microsoft Excel, GraphPadPrism and Partek Genomics Suite software packages were used for statistical calculations and data presentation; p-value< 0.05. An arbitrary cut-off was chosen to describe a gene as being over- or under-expressed in comparisons between two patient cohorts: ≥50 counts by DMGE, a ≥2-fold change in expression (mean of population 1 divided by mean of population 2 ≥2 or ≤2), with a p-value ≤ 0.05, using the student t-test.

## Results

Numerous gene expression profiling studies have been performed during the last two decades on B-CLPD, and CLL in particular, based on microarray technologies. We set out to test a recently developed DMGE method for its potential utility in CLL diagnostics and prognosis determination.

### Genes expressed preferentially by normal B-cells

Pure B cell samples were compared to samples from normal PB. In order for a gene to be considered by normal B cells to be preferentially expressed compared to other blood cells, samples with purified B cells were prepared as described above and compared to samples from normal PB. Out of 290 genes 99 genes fulfilled the criteria of an expression level ≥50 counts, a ≥2-fold change in expression (= mean of the four pure B cell samples divided by the mean of the five normal PB samples ≥2), with a p-value ≤0.05. As expected, this list contained genes coding for B-cell specific surface antigens, namely CD19, CD20 and CD79, as well as for B-cell specific transcription factors, like PAX5 and SOX11, or for immunoglobulin heavy and light chains (Table 1, for the complete list of the 99 genes see S3 Table).

**Table 1:** Genes expressed preferentially by B cells.

Genes	Mean	Mean	Ratio	p-value
	normal PB	pure B cells	pure B/normal PB	
**Surface Markers**				
CD268/BAFF-R	940.25	39402.54	41.9	0.001
CD83	3282.52	136406.91	41.6	0.033
CD79A	3447.71	119505.02	34.7	0.003
CD69	5409.08	181679.96	33.6	0.010
CD23/FCER2	226.31	7362.71	32.5	0.000
CD20	2112.32	66914.33	31.7	0.000
CD22	824.90	23927.76	29.0	0.002
CD19	607.52	17177.12	28.3	0.003
CD40/ TNFRSF5	541.65	12031.25	22.2	0.000
CD200	142.54	3101.54	21.8	0.002
CD79B	181.90	3672.57	20.2	0.039
CD267/TACI	167.24	2259.35	13.5	0.004
CD180	443.95	3135.11	7.1	0.000
CD70/ CD27 ligand	67.95	476.84	7.0	0.007
CD32/FCGR2B	1652.00	6086.44	3.7	0.023
CD81	4430.14	16162.38	3.6	0.007
CD124/IL4R	4546.82	14900.94	3.3	0.000
CD24	919.00	2537.83	2.8	0.013
CD150	288.06	788.85	2.7	0.009
CD71/TFRC	2572.30	6537.91	2.5	0.030
CD74	51335.01	121141.05	2.4	0.000
CD38	496.21	1108.49	2.2	0.048
**Transcription Factors**				
SOX11	1.48	128.09	86.7	0.011
PAX5	267.77	10912.48	40.8	0.000
EBF1	9.86	368.40	37.4	0.021
IRF4	1106.86	7649.87	6.9	0.024
JUN	6269.40	28921.65	4.6	0.000
BCL2	1728.64	7690.48	4.4	0.003
MYC	1640.93	5322.69	3.2	0.000
**Immunglobulin genes**				
IGHD	1071.88	23185.99	21.6	0.000
IGHM	10313.33	198106.21	19.2	0.003
kappa	11354.34	70720.31	6.2	0.000
lambda	17487.73	64435.64	3.7	0.002

Listed are genes with expression values > 50, a pure B cell/normal PB ratio > 2, with a p-value < 0.05.

Fold changes with values of 20–40 corresponded to the ratio of B cells present in the pure B cell and peripheral blood samples (>95% versus 2–3%, respectively) and were found typically for B cell specific genes, like CD19, CD20 and CD22. Fold changes with values <20 correspond to genes which are expressed not only by B cells, but also by other peripheral blood cells, or to genes, for which the hybridization kinetics were not optimal.

### Genes expressed preferentially in CLL samples compared to normal blood cells

**Fig 1:**
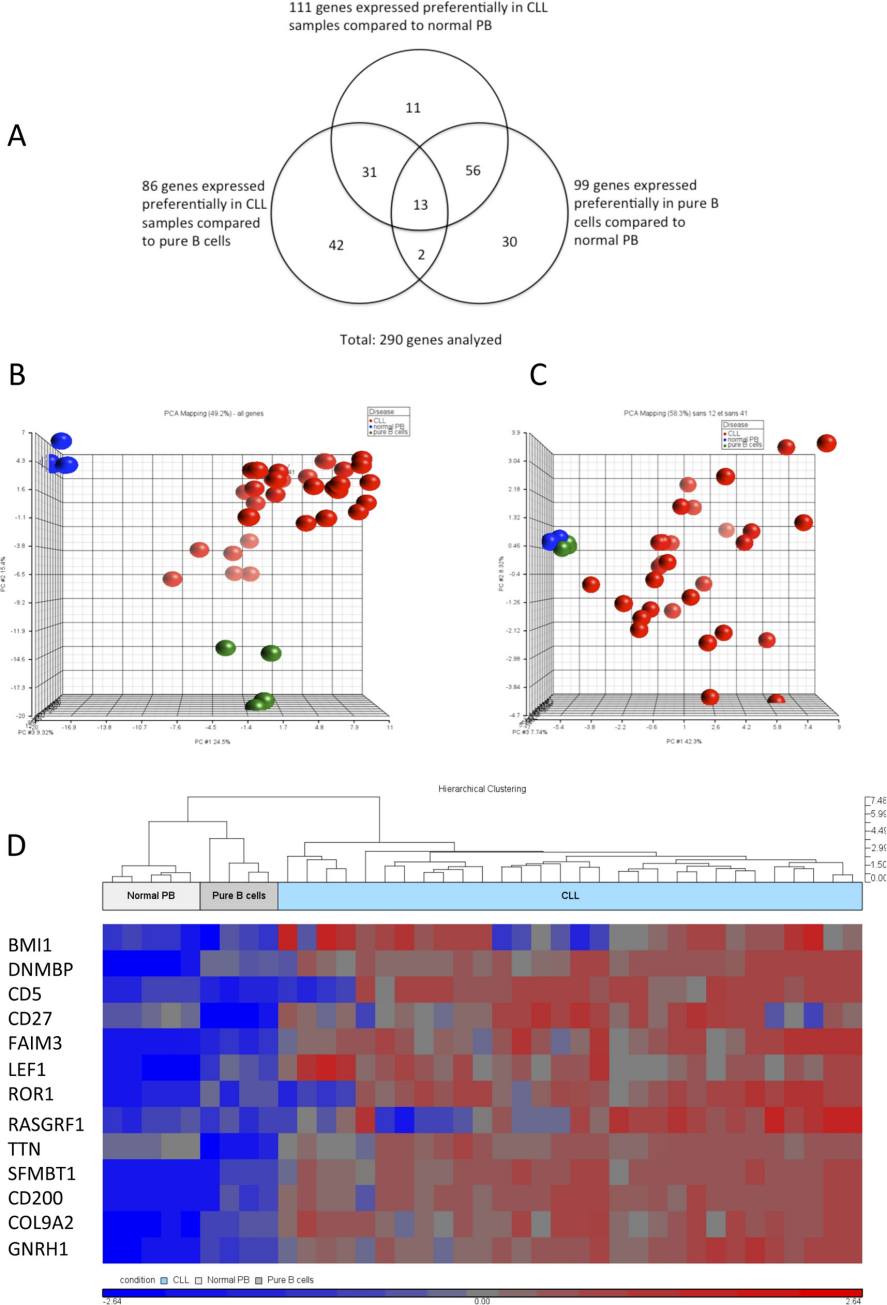
Analysis of the expression of 290 genes in normal PB, pure B cell and CLL samples. (A) Venn diagram of genes expressed preferentially in the different sample groups (normal PB, n = 5; pure B cells, n = 4; and CLL samples, n = 30). Genes were considered preferentially expressed by one sample group, if they showed an expression level ≥50 counts and a ≥2-fold difference in expression levels between the 2 groups, with a p-value ≤0.05. (B) Samples from PB (n = 5), B cell samples (n = 4), and samples from CLL patients (n = 30) were analyzed by PCA, based on the results of the differential expression of 290 genes. (C) PCA analysis on the same samples as in (b), but using a restricted set of 44 genes relevant for this purpose according to their differential expression in CLL, B cells and normal blood. (D) Heat map of normal PB, pure B cells, and CLL samples, analyzed with thirteen genes overexpressed homogenously (CV<5%) in all CLL samples compared to normal PB and pure B cells. Unsupervised analysis shows a perfect clustering of the CLL samples compared to the normal samples.

A panel of 30 CLLs, with a mean of 80% malignant B cells/sample (range: 58–100% per sample) and characterized for their typical cell surface phenotype, and the presence or absence of IgVH mutations, was used for this study. In order to find among the 290 gene list genes that could differentiate most effectively between CLL and normal samples, we compared the mean of all gene counts from the 30 CLL samples to the mean from the normal PB samples, and to the mean of the pure B cell samples. As previously, we selected only genes that fulfilled the arbitrary criteria of an expression level ≥50 counts, a ≥2-fold change in expression with a p-value ≤0.05. Fig 1A shows the Venn diagram for the genes expressed preferentially in the three different sample groups.

We obtained a list of 111 genes expressed preferentially in CLL samples compared to normal PB, and of 86 genes compared to pure B cells (S4 Table). 44 genes were common to both lists (Table 2A). Interestingly, this set of 44 genes contained genes well known to be overexpressed in CLL, like CD5, LPL and ROR1, but also kappa, lambda and IgG genes, showing that on a per-cell-basis CLL B cells produce more IgG mRNA than normal B cells.

**Table 2:** List of genes over- or under-expressed specifically by CLL B cells.

A: List of 44 genes expressed specifically by CLL B-cells				B: List of 28 genes specifically underexpressed in CLL compared to pure B cell samples		
Genes	Mean	SD	CV	Genes	Fold change	p-value
					CLL/pure B cell	
ABCA6	3783.3	1942.9	0.51			
ADAM29	482.5	857.1	1.78	BAFF-R/ CD268	0.426	0.0018
AICDA	69.9	143.7	2.06	BANK1	0.224	0.0042
BIK	414.0	426.0	1.03	BIRC 3	0.484	0.0070
BMI1	607.6	202.4	0.33	CD22	0.359	0.0036
BUB1B	152.4	118.6	0.78	CD40/ TNFRSF5	0.481	0.0000
CD200	10131.9	4443.1	0.44	CD69	0.124	0.0105
CD24	9424.4	7446.1	0.79	CD83	0.086	0.0388
CD269/BCMA	1857.3	1201.7	0.65	CHL1	0.097	0.0191
CD27/ TNFRSF7	8532.6	3512.4	0.41	CHL1_v4	0.144	0.0000
CD5	4039.1	1799.7	0.45	CXCR4/ SDF-1R	0.318	0.0116
CHIT1	243.2	162.8	0.67	EBF1	0.031	0.0210
CLLU1	5605.3	12595.8	2.25	EBI3	0.172	0.0019
CNR1/ CB1	357.5	533.3	1.49	EPB41L2	0.133	0.0011
COL9A2	4955.2	2398.9	0.48	SERPINA9	0.033	0.0408
CTLA4	12573.8	11128.3	0.89	IL6	0.001	0.0032
CXCR3	1609.2	849.2	0.53	ITGA4/ CD49d	0.410	0.0000
DMD	3625.7	3727.6	1.03	JAM3/ JAM-C	0.129	0.0001
DNMBP	4539.7	2102.5	0.46	JUN	0.411	0.0002
FAIM3/ Toso	44146.0	20379.2	0.46	LGMN	0.114	0.0249
FCER2/ CD23	15988.5	8133.0	0.51	MMP12	0.019	0.0003
FGF2	707.3	693.6	0.98	MS4A1/ CD20	0.293	0.0001
FGFR1	554.3	784.8	1.42	MYC	0.198	0.0001
FILIP1L	2284.9	1636.2	0.72	NREP	0.280	0.0050
FLT3	406.5	512.4	1.26	RAGE/ MOK	0.451	0.0086
FMOD	17401.2	12667.9	0.73	REL	0.335	0.0005
GNRH1	518.1	232.4	0.45	SOX11	0.189	0.0105
IGFBP4	4858.1	3366.4	0.69	TIMP4	0.011	0.0107
IGHG1 to 4	13373.7	11186.2	0.84	ZFP36	0.426	0.0056
IGSF3	1129.0	692.7	0.61			
IL2RA/ IL2R	2205.9	1462.3	0.66			
kappa	128933.8	120715.3	0.94			
lambda	142654.3	170967.7	1.20			
LEF1	7270.0	2323.7	0.32			
LILRA4	1799.4	2540.1	1.41			
LPL	641.1	681.2	1.06			
RAPGEF3	817.9	457.3	0.56			
RASGRF1	2960.0	1436.7	0.49			
ROR1	2376.8	1107.6	0.47			
Selectin P/ CD62	1067.6	708.9	0.66			
SEPT 10	667.5	1305.7	1.96			
SFMBT1	11065.5	3547.0	0.32			
TTN	7243.9	3010.2	0.42			
WNT3	5240.6	5963.0	1.14			

(A) List of 44 genes expressed specifically by CLL B cells. Expression of mRNA was compared between the mean of 30 CLL samples and the mean of 5 normal PB and 4 pure B cell samples. Listed are those genes that fulfill the following criteria: > 2-fold change in expression between CLL samples and normal PB samples and pure B cell samples; p-value < 0.05; expression level in CLL samples > 50. (B) List of genes underexpressed by CLL samples compared to pure B cell samples. Listed are those genes that fulfill the following criteria: < 0.5-fold change in expression between CLL samples and pure B-cell samples; p-value < 0.05; expression level in pure B cell samples > 50.

As expected, principal component analysis (PCA) performed on all the 290 genes resulted in a clear separation of the samples according to their origin (pure B cells, normal PB or CLL; Fig 1B); restricting the PCA analysis to the 44 genes defined above resulted in a slightly different distribution, with normal PB and pure B cells clustered together, but with all CLL samples clearly separated from them (Fig 1C). A detailed analysis of the expression levels of these 44 genes showed a wide range of coefficients of variation (Table 2A). 13/44 genes were expressed at a highly similar expression level in all CLL samples, with a CV < 0.5 (BMI1, CD200, CD27, CD5, COL9A2, DNMBP, FAIM3, GNRH1, LEF1, RASGRF1, ROR1, SFMBP1, TTN) (S1 Fig). These thirteen genes with the lowest CV can therefore be used as a classifier, which allows unambiguously to separate CLL samples from normal PB samples, being restricted to genes preferentially and homogenously expressed in CLL B cells compared to normal B cells (Fig 1D).

Several genes were also found to be specifically underexpressed in all CLL samples as compared to samples with pure B cells (Table 2B). These genes correspond to genes downregulated in CLL B cells compared to normal B cells, like IL-6, TIMP4 and MMP12, whose mRNA is absent in CLL B cells although normal B cells produce them in high amounts (mean: 1758.9; 92.7; 90.5 copies/sample, respectively).

### Correlation between protein expression and mRNA expression

Flow cytometry allows the analysis of surface and intracytoplasmatic antigens of CLL B-cells. We studied the correlation between some antigens measured by routine flow cytometry (i.e.: CD19, CD20, CD5, CD23, CD38, CD200, kappa, lambda, ZAP70) and mRNA counts obtained by the nCounter measurements (Table 3). These expression levels corresponded to the protein expression levels detected by flow cytometry. In all the samples the CLL B cells strongly expressed CD5, CD23, CD43 and CD200 on their surface, whereas CD20 expression was found decreased compared to normal B cells.

**Table 3:** Quantification of mRNA transcripts for surface proteins.

Genes	Mean	Mean	Mean	Ratio CLL/normal PB	Ratio CLL/pure B cells	p-value	
	normal PB	pure B cells	CLL				
CD43	1886.17	17.20	1253.7	0.7	72.9	0.000	increased expression compared to normal B cells
CD5	1782.16	250.99	4039.1	2.3	16.1	0.000	increased expression compared to normal B cells
CD200	142.54	3101.54	10131.9	71.1	3.3	0.000	increased expression compared to normal B cells
FCER2/ CD23	226.31	7362.71	15988.5	70.6	2.2	0.000	increased expression compared to normal B cells
CD79B	181.90	3672.57	4665.0	25.6	1.3	0.421	equal expression compared to normal B cells
CD19	607.52	17177.12	19164.6	31.5	1.1	0.480	equal expression compared to normal B cells
CD38	496.21	1108.49	860.8	1.7	0.8	0.760	equal expression compared to normal B cells
CD79A	3447.71	119505.02	64178.8	18.6	0.5	0.016	decreased expression compared to normal B cells
CD22	824.90	23927.76	8597.4	10.4	0.4	0.004	decreased expression compared to normal B cells
MS4A1/ CD20	2112.32	66914.33	19628.9	9.3	0.3	0.000	decreased expression compared to normal B cells

**Fig 2:**
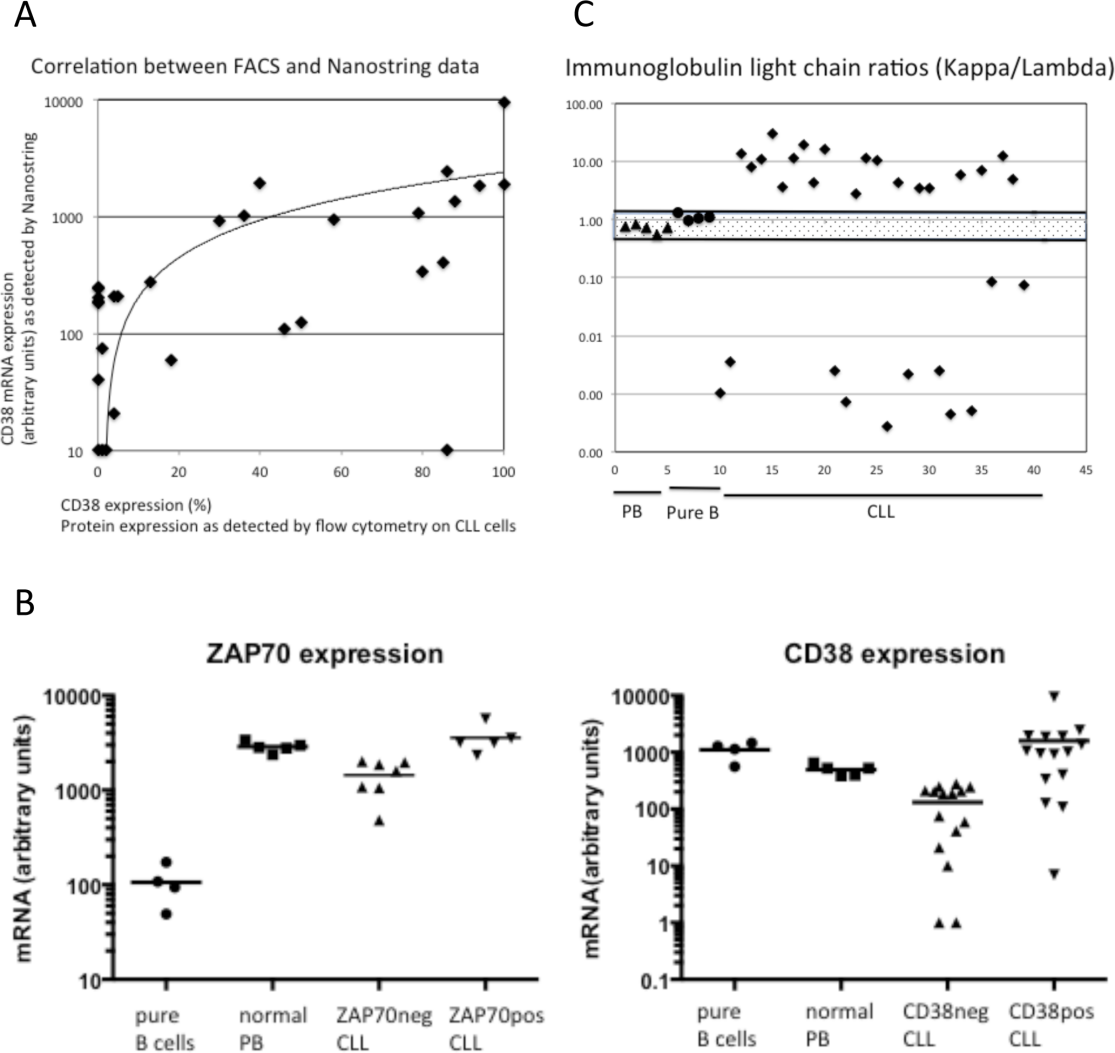
Correlation between mRNA and protein expression. **(A)** Correlation between CD38 protein expression, as measured by flow cytometry (% positive CLL B cells) and CD38 mRNA counts, as measured by the nCounter (arbitrary units). (B) Quantification of CD38 and ZAP70 mRNA counts in normal peripheral blood (PB), pure B cells, and in CLL B cells. CLL B cells were analyzed for ZAP70 and for CD38 expression by flow cytometry and then grouped for the mRNA determination, according to presence or absence of these two antigens. (C) Immunoglobulin light chain ratios in 30 CLL patient samples (rhombi) and in normal B cells (triangles: normal PB samples; circles: pure B cell samples). The mean +/- 2SD interval for ratios from polyclonal normal B cells and normal PB is shown (mean 0.89; SD = 0.22; small dots).

Correlating the CD38 mRNA counts with the results from flow cytometry (S1 Table) resulted in a correlation coefficient of 0.53, similar to values of correlation coefficients found in a previous study on surface antigens in acute myeloid leukemia (AML) blasts [9] (Fig 2A). We then compared the mRNA counts in the cohort of CD38pos CLL patients with the CD38neg cohort, and the ZAP70pos cohort to the ZAP70neg one: the expected results were found, with CD38pos and ZAP70pos B-cell samples exhibiting higher mRNA counts then the corresponding negative samples (Fig 2B). Interestingly, the comparison with normal B cells gave different results for CD38 and ZAP70: normal B cells, although expressing low surface CD38 protein, showed higher CD38mRNA counts than the CD38neg CLL samples, but lower ZAP70 mRNA counts than the ZAP70neg CLL samples (Fig 2B).

Analysis of the kappa/lambda ratio at the mRNA and protein level showed a 100% correlation between flow cytometry results and mRNA counts, and all CLL cases, which were monoclonal by cytometry also showed abnormal ratios in their mRNA counts (Fig 2C).

### Analysis of mutated and unmutated cases of CLL

One of the most significant prognostic factors identified in CLL that ultimately ties to the biology of disease is the mutational status of the variable region of the immunoglobulin heavy chains. We determined the mutation status in our cohort of patients and found 11 patients with mutated and 17 patients with unmutated IgVH, 2 patients were borderline (S1 Table). In order to determine which genes were correlated with the mutation status, we compared mutated to unmutated samples and listed genes either overexpressed in mutated versus unmutated, or in unmutated compared to mutated samples (Table 4). 24 genes were found to be differentially expressed: nineteen genes were overexpressed in unmutated, and five in mutated samples. Among the differentially expressed genes were nine genes from the 44-gene-list, which we used to distinguish CLL from normal samples, as well as several genes described in the literature: CD38, ZAP-70, LPL, etc. ([4],[16],[17],[18]; see also Table 4).

**Table 4:** List of genes distinguishing mutated from unmutated CLL samples.

**Genes upregulated in unmutated IgVH**							
Genes	mutated		unmutated		Ratio	p-value	Literature Reference
	IgVH		IgVH		unmut/mut		
	Mean	SD	Mean	SD			
SEPT 10	10	20	1172	1556	121.0	0.00866	[19]
AICDA	1	0	121	174	106.5	0.01562	[20,21]
LDOC1	7	14	368	233	55.4	0.00001	[22] [23]
FARP1	3	4	54	83	18.4	0.02412	
LPL	91	89	1072	620	11.7	0.00001	[24] [25] [18] [23] [26]
CNR1	50	71	582	616	11.6	0.00413	[27]
CD38	137	119	1429	2119	10.4	0.02506	[4] [17]
DMD	1421	1718	5379	3931	3.8	0.00115	[19] [23]
CEACAM1	194	165	590	540	3.0	0.02355	
CRY1	501	571	1380	450	2.8	0.00007	[28] [29]
ZAP70	1341	563	3482	1209	2.6	0.00001	[4] [17] [16]
ITGA4	600	318	1529	1079	2.5	0.00632	[30]
TCL1A	15285	14608	38680	18435	2.5	0.00048	[19]
CD26	60	63	151	105	2.5	0.00429	[31] [32]
CHIT1	135	100	325	157	2.4	0.00045	
IGHM	145269	131768	333881	138491	2.3	0.00494	[33]
EPB41L2	136	116	305	293	2.2	0.03937	
VPREB3	2530	1420	5505	1873	2.2	0.00265	
ABCA6	2295	1010	4643	1812	2.0	0.00351	
**Genes upregulated in mutated IgVH**							
	mutated		unmutated		Ratio	p-value	
	IgVH		IgVH		mut/unmut		
	Mean	SD	Mean	SD			
CTLA4	18370	13614	8503	6686	2.2	0.03908	
RARA	3398	3056	1185	586	2.9	0.03850	
LGMN	114	94	38	38	3.0	0.03412	
CD150	1665	1117	490	326	3.4	0.00781	[34] [35]
ADAM29	1061	1091	66	259	16.0	0.00803	[24] [19]

Genes with an expression level > 50, and a ratio mut/unmutated or unmutated/mutated > 2 with a p-value < 0.05 are shown; References from the literature for each cited gene are given, when available.

**Fig 3:**
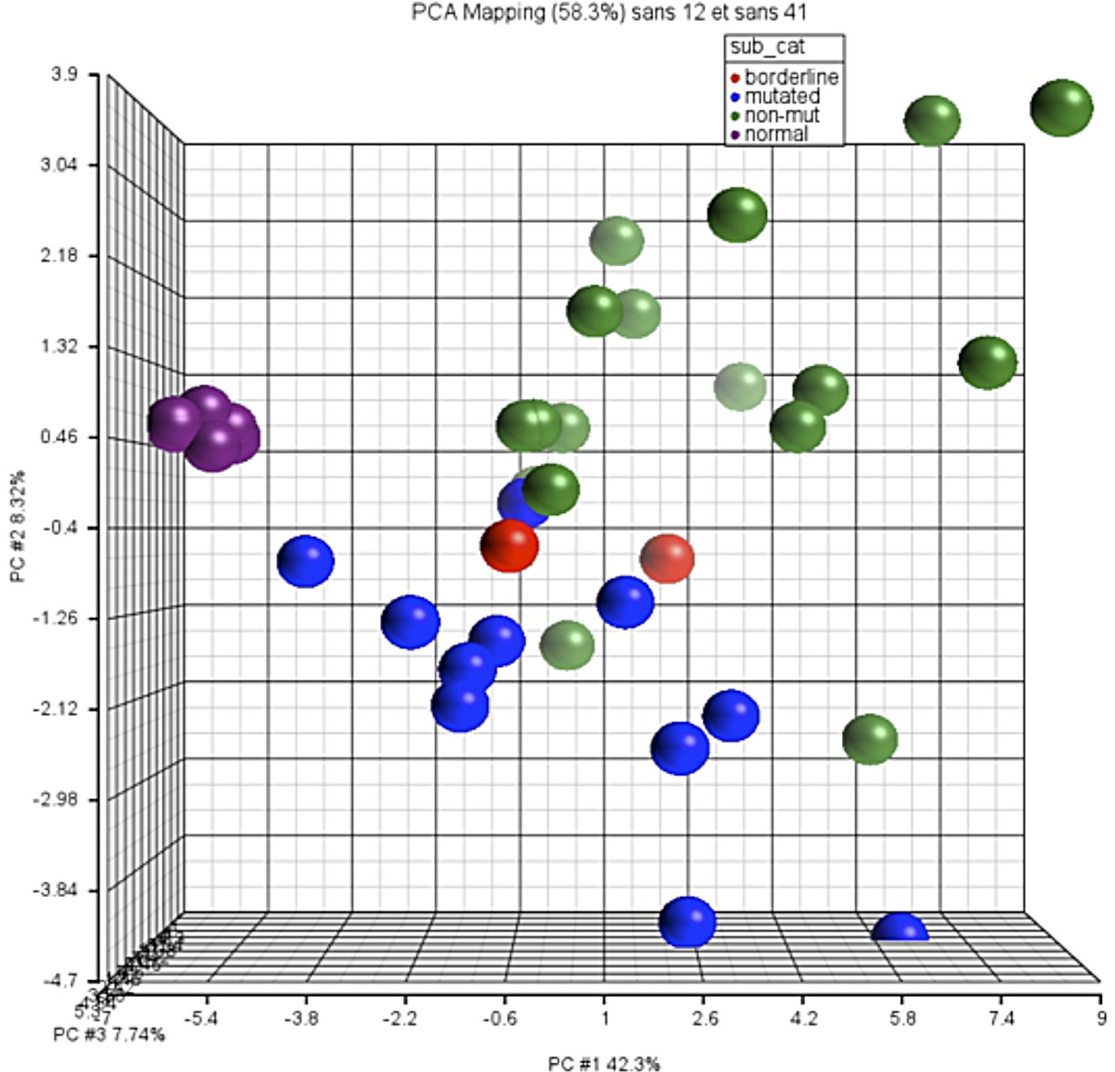
Characterization of different types of CLL samples using PCA analysis. Samples from PB (n = 5), B cell samples (n = 4), and samples from CLL patients (n = 30) were analyzed by PCA, based on the results of the differential expression of 44 genes. Mutated CLL cases are shown in blue (n = 11), unmutated in green (n = 17), and borderline cases in red (n = 2).

This 24-gene panel called “LymphCLL Mut” allowed a clear distinction between both types of CLL (Fig 3; S2 Fig).

Total light chain production was increased in mutated vs unmutated samples (185207 counts vs 324812 counts; p = 0.008).

### Analysis of LDOC1 expression

**Fig 4:**
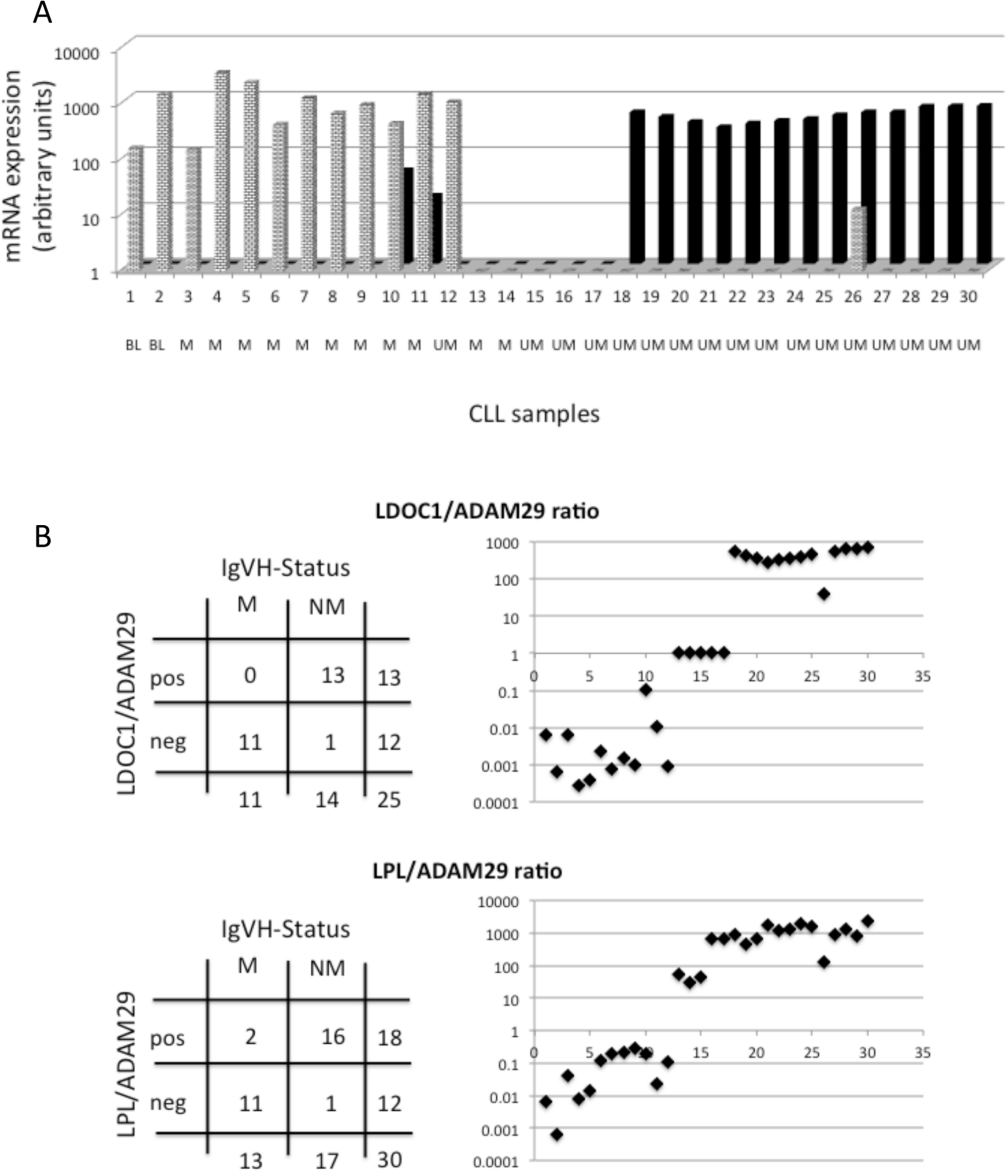
Analysis of LDOC1 expression. (A) LDOC1 mRNA expression was measured in 30 CLL samples and samples ordered according to absent/low (No 1–17) or high expression (No 18–30); black columns. ADAM29 mRNA expression shows an inverse pattern (No 1–12); hashed columns. Mutation status is noted below (BL = borderline; M = mutated; UM = unmutated IgVH) (B) Analysis of LDOC1/ADAM29 and LPL/ADAM29 ratios.

LDOC1 mRNA has been reported to be highly expressed in aggressive cases of CLL and to correlate with IgVH mutation status and with prognosis [22]. When we analyzed the mRNA expression in our 30 CLL cases, we found indeed a dichotomic distribution, completely different from the homogenous distribution, which we described in the thirteen genes used for the CLL classifier (S1 Fig). Interestingly, when we looked among all the 290 genes analyzed, only eight genes were found to correlate with LDOC1 expression: six genes with a positive correlation (SEPT10, LPL, CD26, EPB41L2, CXCR6, CRY1) and two genes with a negative correlation (ADAM29, CD150; Table 5; S3 Fig). ADAM29 was exclusively expressed in samples with absent/low LDOC1 expression and vice versa (Fig 4A). Superficially, this expression pattern corresponded to the IgVH mutation status of these samples, but a closer inspection yielded a group of five samples with absence of both LDOC1 and ADAM29 mRNAs (two mutated and three unmutated cases). The LDOC1/ADAM29 ratio clearly reflects this separation into three different groups of samples. Interestingly, in a previous report Oppezzo *et al* have published the LPL/ADAM29 ratio as a surrogate marker for IgVH status [24]. Comparing this ratio in our samples to the IgVH status showed concordance in 27/30 samples; the three discordant samples corresponded to samples with absent LDOC1 or ADAM29 expression (Fig 4B).

**Table 5:** LDOC1 expression in CLL samples.

Genes	Mean	Mean	Ratio	p-value
	LDOC1 neg	LDOC1 pos	LDOC1 pos/neg	
LDOC1	1	483	483.4	0.0000
SEPT 10	68	1365	20.2	0.0138
LPL	154	1331	8.6	0.0000
CD26/ DPP4	45	162	3.6	0.0023
EPB41L2	132	353	2.7	0.0280
CXCR6	33	89	2.7	0.0374
CRY1	551	1404	2.5	0.0001
			**Ratio**	
			LDOC1 neg/pos	
CD150	1373	462	3.0	0.0086
ADAM29	832	2	451.3	0.0096

CLL samples were separated into those highly expressing LDOC1 and those with absent/low expression. Listed are those genes that fulfill the following criteria: >2-fold change in expression between LDOC1 pos samples compared to LDOC1 neg samples, with a p-value < 0.05 or >2-fold change in expression between LDOC1 neg samples compared to LDOC1 pos samples, with a p-value < 0.05.

### Validating the CLL classifier

**Fig 5:**
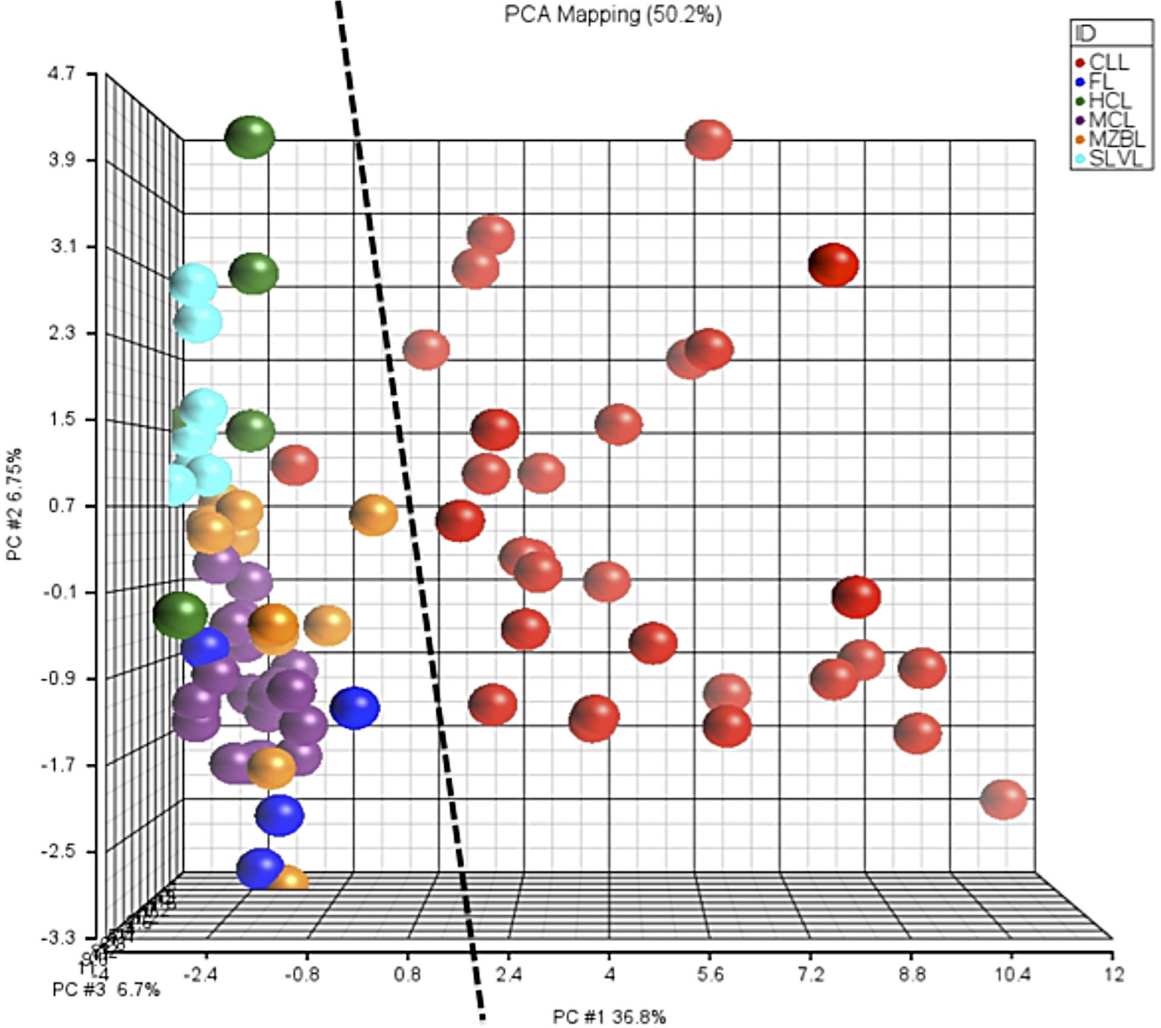
Comparison of CLL to other B-CLPD using PCA. Samples from CLL patients (n = 30) and of other B-CLPD (n = 51) were analyzed by PCA, based on the results of the differential expression of the selected 44 genes.

In order to develop a clinically useful classifier, CLL samples not only have to be distinguished unambiguously from normal PB samples, but also from other lymphoma subtypes. We therefore tested the 44 genes found to distinguish CLL from normal PB samples on a series of 51 patients with different B-CLPD, i.e., MCL, MZL, FL and HCL. The PCA analysis showed a clear separation of the CLL from all the other B-CLPD samples, with the exception of one confirmed CLL case, which was misdiagnosed (Fig 5). None of the B-CLPD samples was misdiagnosed as a CLL.

## Discussion

In the present work we describe the use of DMGE, a recently developed technique for the quantitative and parallel analysis of hundreds of mRNAs, in a cohort of 30 CLL patients. Starting from a set of 290 genes with preferential expression in B cells and CLL cells described in previously published reports, we were able to establish lists of genes, with preferential expression in normal and in CLL B cells, respectively, and which allowed distinguishing unambiguously CLL samples from normal PB samples and CLL B cells from normal B cells. Restricting these gene lists to genes expressed homogenously by all CLL samples, independent of chromosomal abnormalities, yielded a classifier, based on the analysis of only 13 genes. Applying this classifier in an unsupervised analysis of our cohort resulted in a perfect separation of all 30 CLL samples. Adding kappa/lambda ratios to the classifier will certainly increase its discriminative power, since our results show for all cases a clear distinction between polyclonal samples (normal PB and sorted normal B cells) and CLL samples with essentially monoclonal B cell populations.

In a previous study, we have already used DMGE in acute myeloid leukemia (AML) to correlate leukemic blast mRNA expression with surface antigens determined by flow cytometry [9]. The present study confirms close correlation between flow cytometry results and DMGE analysis for some surface proteins.

The DMGE technique also allows the study of genes with prognostic relevance in parallel with the 13 gene diagnostic classifier, using a single assay. Interestingly, these genes fall broadly into two categories: those expressed with wide variations in different samples (up to 10^4^ difference in mRNA expression; e.g.: LILR4 and CLLU1), and those with a present/absent, dichotomic pattern (e.g.: LDOC1, LPL, ADAM29).

Analysis of the IgVH mutation status is widely used to distinguish patients with a good from those with a bad prognosis. Several surrogate markers have been described in the literature and shown to correlate with the IgVH mutation status. We could confirm most of them, such as ZAP70, CD38, LPL and LDOC1 (Table 4). On the contrary, we did not find any differential expression for the following genes FCRL2 (p = 0.08) and HS1 (p = 0.10), also reported to vary between mutated and unmutated CLL samples [36], [37]. In an unsupervised analysis 28/30 (93%) CLL samples were correctly classified using the “Lymph CLL Mut” classifier based on 24 genes with a differential expression between IgVH mutated and unmutated cases.

The LPL/ADAM29 ratio has already been described previously to constitute a surrogate marker for the IgVH mutation status [24], and also to be related to prognosis [38]. Whereas this ratio distinguishes two different types of CLL samples, the determination of the LDOC1/ADAM29 ratio allowed distinction of 3 subclasses: IgVH mutated with high expression of ADAM29, unmutated samples with high expression of LDOC1, and a third category (mixed mutated and unmutated samples) without expression of LDOC1 and ADAM29. This third group did not show any common IgVH usage or chromosomal abnormalities. Future studies have to tell us whether there is any clinical significance or any existing correlations between this category and prognosis.

In our final analysis we tested the 44-gene signature, which differentiated CLL from normal PB samples, on a set of 51 samples from patients with various common B-CLPD. Similar to the flow cytometric RMH score our “LymphCLL Diag” molecular classifier distinguished CLL from other B-CLPD with high sensitivity and specificity (97% and 100%, respectively). Associating the “LymphCLL Diag” gene panel with the “LymphCLL Mut” panel, the kappa/lambda ratio and the LDOC1/ADAM29 ratio, a complete diagnostic and prognostic procedure could be performed in one single “ready to use” assay, based on a panel of 61 genes.

Several of the laboratory analyses described for diagnostic and prognostic purposes in CLL are time- and labor- intensive and not well suited for routine testing in most clinical laboratories. One example is the determination of the IgVH mutational status, which is rather expensive, needs specialized know-how, and is currently only performed in a restricted number of laboratories under the expertise of molecular biologists [39]. Threshold levels are arbitrary (in most reports > 2% are considered mutated) and a grey zone exists [39].

Another example is the ZAP70 expression analysis by flow cytometry, which has been largely abandoned due to difficulties in standardization [40] [41] or the ZAP expression analysis by RT-PCR, which requires purification of B cells prior to the assay [39], rendering this approach unsuitable for routine diagnostics.

The new sequencing technologies also hold the promise to give valuable data for prognosis determination of CLL patients, most notably TP53, *NOTCH1*, *ATM*, *SF3B1* and *BIRC3* mutations [42] [43]. Mutations in these genes occur in approx. 2%-17% of CLL patients at diagnosis and the prognostic importance of some of them have already been studied in prospective trials [44] [45]. Whether this information is complementary to established prognostic factors and results from mRNA and gene expression studies like ours have ideally to be investigated in large future prospective and comparative trials. Although already widely used for research purposes deep sequencing techniques are not yet used in routine laboratories and the expensive, labor-intensive technology and bioinformatically complex softwares will make this transfer challenging.

With the development of DMGE a new technique has arrived, which allows genetic profiling with the parallel analysis of hundreds of mRNAs by a technically extremely simple method. DMGE has a short turn-around time of < 2 days, needs minimal hands-on-time for technicians and is much less costly than whole gene expression profiling or deep sequencing. Moreover, this technique allows for automated data-analysis, and has a read-out, which is intuitive and does not need complicated bio-informatics tools for the analysis or interpretation. By focusing our approach on the analysis of a highly selected set of genes expressed preferentially by B cells, we could obtain signatures from the analysis of whole blood samples, rendering an additional B cell purification step unnecessary.

The parallel quantitative analysis of tens to hundreds of mRNAs allows the integration of several diagnostic and prognostic factors in one assay, contrary to numerous studies from the past, which have only analyzed one or two factors at a time. It should be therefore ideally suited for large trials aiming at the comparison of multiple factors in many different patient samples and for molecular characterization of cases without available living cells for flow cytometry, such as cDNA or FFPE. Smaller labs could also profit from this approach for routine diagnostics based on an automated data analysis.

To fully appreciate the clinical usefulness and discriminative power of this approach, prospective studies with a much larger number of CLL samples will have to be performed in the future, including samples with other B-CLPD and reactive/inflammatory conditions. Additional prognostic markers can be easily incorporated to the classifier and then be studied simultaneously in clinical trials, but also in routine diagnosis. Integrating information from gene profiling studies with results from genomic mutation and NGS analyses should ultimately lead to better prognostication schemes for patients.

## Supporting Information

S1 Fig Comparison of mRNA counts from normal PB, pure B cell and CLL samples. Shown are 13 selected genes from the 44-gene list with a low CV < 0.5.
(TIF)

S2 Fig Heatmap of 30 CLL samples, analyzed with the 24-gene classifier for the recognition of mutated vs unmutated samples. (TIF)

S3 Fig Heatmap of CLL samples based on analysis of genes correlated with expression of LDOC1 mRNA (unsupervised analysis). (TIF)

S1 Table Patient characteristics. 30 patients with a diagnosis of typical CLL were included in the study. Flow cytometry was used to determine the % of malignant CLL B cells/sample, and the % of CD38+ CLL B-cells; values ≥20% were considered positive. Mutation status of the IgVH was determined as described in Material and Methods section. ND = not done.
(XLS)

S2 Table Probes for Nanostring analysis. 290 genes were chosen to constitute the CodeSet for this work, plus nine housekeeping genes.
(XLSX)

S3 Table mRNA expression of 290 genes for all 30 CLL patient samples. (XLSX)

S4 Table List of genes expressed preferentially by CLL samples compared to normal PB (A) and pure B cell samples (B). (XLSX)
