# Supplementary figures and images for: Developing Molecular Signatures for Chronic Lymphocytic Leukemia
Source: PLoS One. 2015 Jun 5;10(6):e0128990. doi: 10.1371/journal.pone.0128990 (PMC4457530; doi:10.1371/journal.pone.0128990)

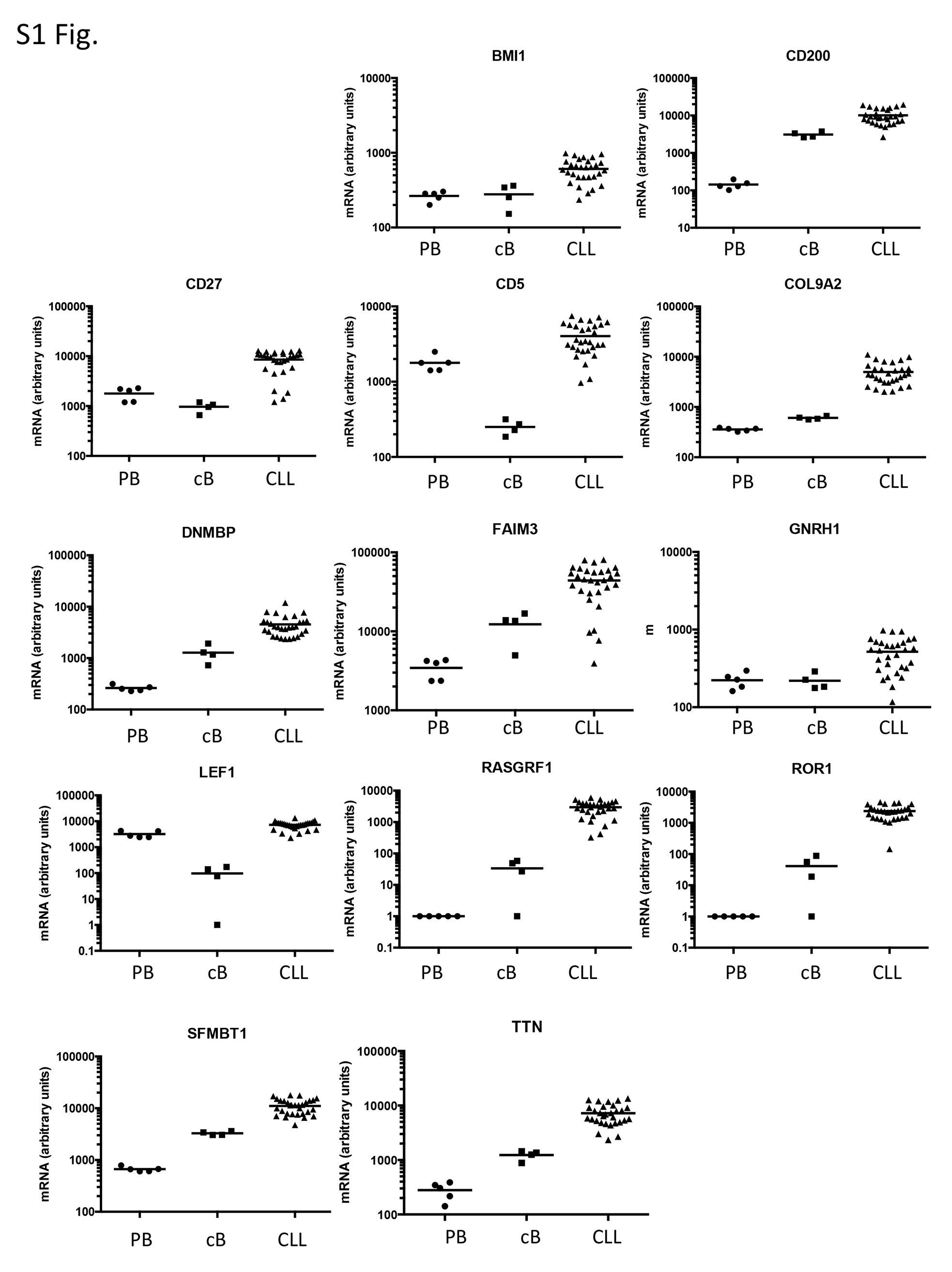

Supplement: S1 Fig — Shown are 13 selected genes from the 44-gene list with a low CV < 0.5. (TIF) [file pone.0128990.s001.tif]

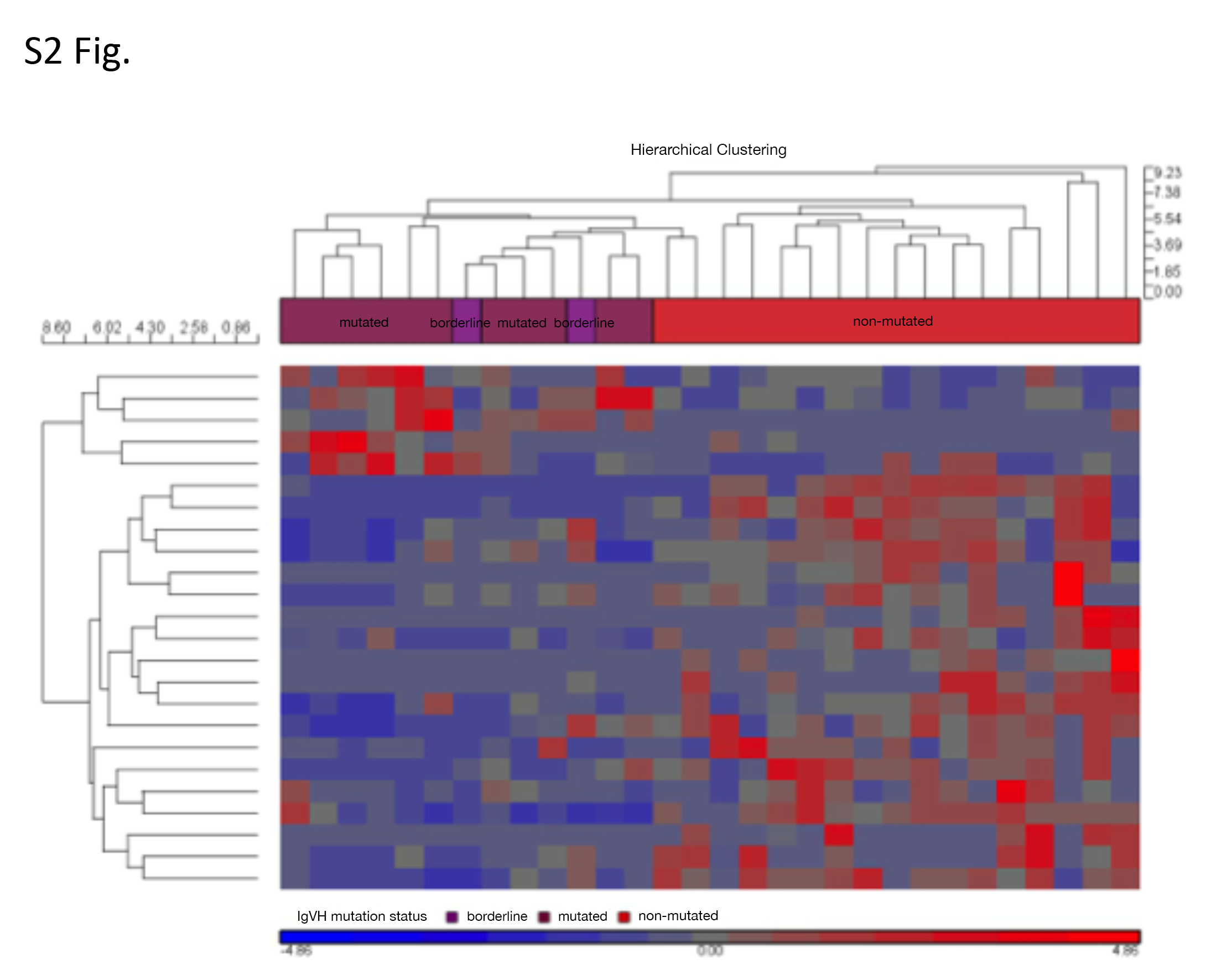

Supplement: S2 Fig — (TIF) [file pone.0128990.s002.tif]

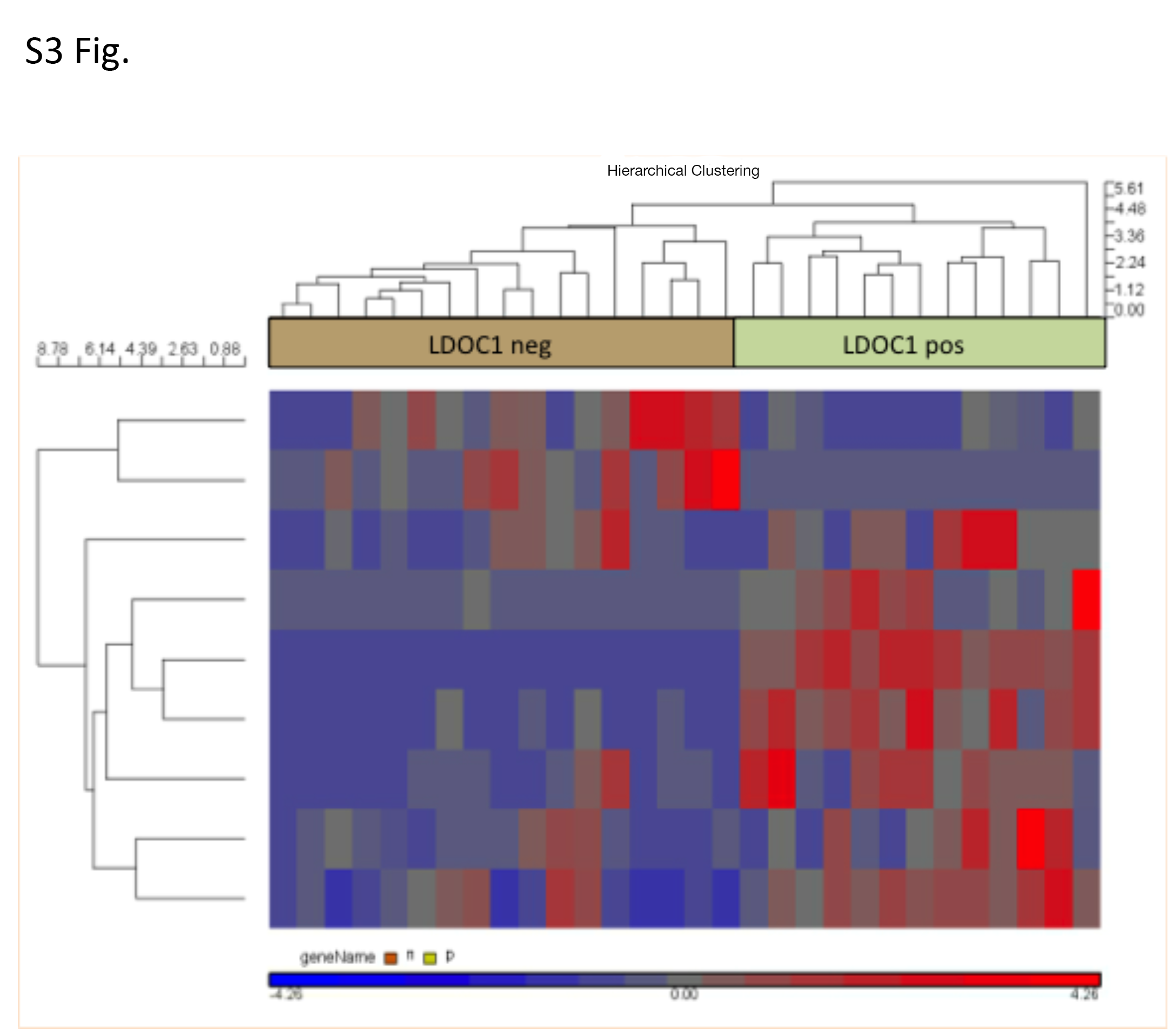

Supplement: S3 Fig — (TIF) [file pone.0128990.s003.tif]
